# Genome-wide analysis of differential RNA editing in epilepsy

**DOI:** 10.1101/gr.210740.116

**Published:** 2017-03

**Authors:** Prashant Kumar Srivastava, Marta Bagnati, Andree Delahaye-Duriez, Jeong-Hun Ko, Maxime Rotival, Sarah R. Langley, Kirill Shkura, Manuela Mazzuferi, Bénédicte Danis, Jonathan van Eyll, Patrik Foerch, Jacques Behmoaras, Rafal M. Kaminski, Enrico Petretto, Michael R. Johnson

**Affiliations:** 1 Division of Brain Sciences, Imperial College Faculty of Medicine, London W12 0NN, United Kingdom;; 2 Centre for Complement and Inflammation Research (CCIR), Imperial College London, London W12 0NN, United Kingdom;; 3 Institut Pasteur, Unit of Human Evolutionary Genetics, Paris 75015, France;; 4 Duke-NUS Medical School, Singapore 169857, Republic of Singapore;; 5 Neuroscience TA, UCB Pharma, 1420 Braine-l'Alleud, Belgium

## Abstract

The recoding of genetic information through RNA editing contributes to proteomic diversity, but the extent and significance of RNA editing in disease is poorly understood. In particular, few studies have investigated the relationship between RNA editing and disease at a genome-wide level. Here, we developed a framework for the genome-wide detection of RNA sites that are differentially edited in disease. Using RNA-sequencing data from 100 hippocampi from mice with epilepsy (pilocarpine–temporal lobe epilepsy model) and 100 healthy control hippocampi, we identified 256 RNA sites (overlapping with 87 genes) that were significantly differentially edited between epileptic cases and controls. The degree of differential RNA editing in epileptic mice correlated with frequency of seizures, and the set of genes differentially RNA-edited between case and control mice were enriched for functional terms highly relevant to epilepsy, including “neuron projection” and “seizures.” Genes with differential RNA editing were preferentially enriched for genes with a genetic association to epilepsy. Indeed, we found that they are significantly enriched for genes that harbor nonsynonymous de novo mutations in patients with epileptic encephalopathy and for common susceptibility variants associated with generalized epilepsy. These analyses reveal a functional convergence between genes that are differentially RNA-edited in acquired symptomatic epilepsy and those that contribute risk for genetic epilepsy. Taken together, our results suggest a potential role for RNA editing in the epileptic hippocampus in the occurrence and severity of epileptic seizures.

Epilepsy is a common, serious disease characterized by unprovoked and spontaneously recurring epileptic seizures. Approximately 2% of the world's population is affected by epilepsy at some time in their lives (Hauser et al. 1993; Prinz 2008). Epilepsy can develop following brain injury or as a consequence of genetic predisposition (Speed et al. 2014). Large-scale mapping of common and rare gene variants has identified several genes that confer risk for genetic epilepsy (Allen et al. 2013; EuroEPINOMICS-RES Consortium; Epilepsy Phenome/Genome Project; Epi4K Consortium 2014; International League Against Epilepsy Consortium on Complex Epilepsies 2014), but the role of sequence variation arising as a result of post-transcriptional events such as RNA editing (RE) remains largely unexplored in epilepsy.

RNA editing can be defined as any post-transcriptional event other than splicing that alters nucleotide composition of a transcript, compared to the corresponding DNA template. In mammals, the main types of RE include the conversion of adenosine to inosine (A-to-I) where inosine is translated as if it were guanosine (i.e., A-to-G), and the conversion of cytosine to uracil (C-to-U) where uracil is translated as if it were thymine (C-to-T). These base-specific changes to RNA result from site-specific deamination of nucleotides catalyzed by ADARs (adenosine deaminases that act on RNA) and APOBEC1, the latter belonging to the APOBEC cytidine deaminases (Roberts et al. 2013). RE is a dynamically regulated process that in coding regions could lead to alteration in protein function and represents an important mechanism to expand and diversify functions of the protein repertoire (Wahlstedt et al. 2009). As an example, within the neural serotonin receptor *HTR2C* gene transcripts, five RE sites have been reported in close proximity to each other, which produce a diverse repertoire of 28 mRNAs and 20 protein isoforms (Du et al. 2006). RE may also occur within noncoding sequence such as within the 3′ untranslated region (UTR) of target transcripts, thereby affecting gene function through changes in transcript stability and/or translational efficiency (Liu et al. 2014).

The A-to-I type of RE has been reported as the most abundant conversion, with over 16,000 RNA-edited sites in whole brain tissue described (Lee et al. 2015) and with extensive evolutionary conservation of both the edited sites and the level of the RE at those sites (Danecek et al. 2012). Studies based on candidate gene analysis have suggested a potential role for RE in several brain disorders including epilepsy, Alzheimer's disease, Huntington's disease, depression, schizophrenia, and amyotrophic lateral sclerosis (Akbarian et al. 1995; Maas et al. 2006; Farajollahi and Maas 2010). However, to date, no large-scale genome-wide analysis of differential RNA editing in the diseased brain has been carried out, potentially due to an absence of suitable control brain tissue from healthy human subjects as well as because of concerns related to systematic technical biases arising from mapping and sequencing ambiguities (Danecek et al. 2012).

In this study, we performed a genome-wide differential RE association study (GWRAS) of epilepsy using a mouse model of acquired symptomatic epilepsy. Animal models of epilepsy have played a fundamental role in advancing our understanding of the basic mechanisms underlying ictogenesis and epileptogenesis (Loscher 2011) and offer the unique advantage of allowing the analysis of matched disease and healthy control brain tissue. Here, we used a mouse model of temporal lobe epilepsy (TLE), where the mice develop spontaneous recurrent seizures (SRS) (i.e., epilepsy) a few weeks following the induction of status epilepticus (SE) by an injection of pilocarpine (Mazzuferi et al. 2013). As well as manifesting SRS, these mice also reflect several of the behavioral and cognitive disturbances associated with TLE in humans (Groticke et al. 2007).

## Results

### Study design

**Figure 1.**
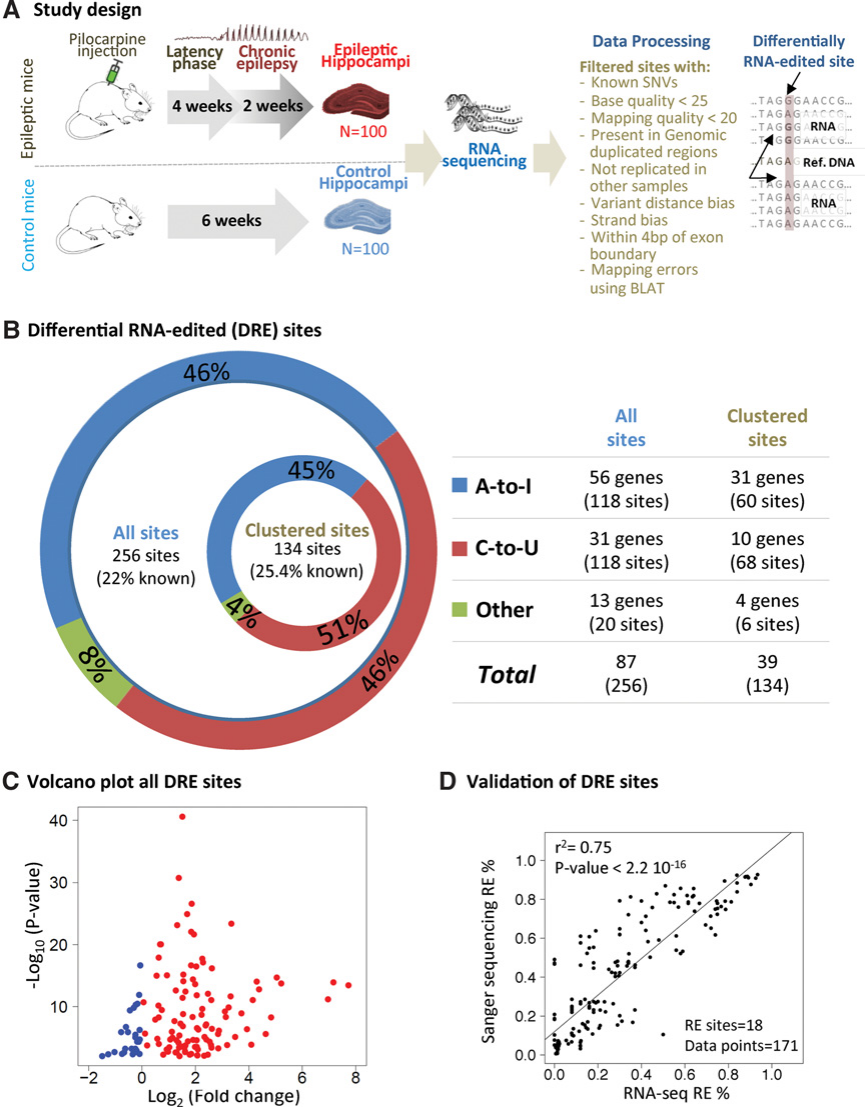
(*A*) Summary of study design for detecting significant differential RNA editing events associated with epilepsy. (*B*) The figure summarizes the results for the different categories of differential RNA editing analyzed. These categories were generated using two approaches: (1) predicted RNA-editing sites, represented in the *outer* circle, and (2) predicted and clustered RNA-edited sites, represented in the *inner* circle and table. The percentages were calculated with respect to unique genes. The *outer* circle refers to “all” sites, while the *inner* circle refers to “clustered” sites. A-to-I and C-to-U editing are represented in blue and red, respectively, while all other sites are represented in green. (*C*) Volcano plot summarizing mean RNA-editing percentages differences in epileptic and control mice and their significance levels. (*D*) Concordance between RNA-editing events detected using RNA-seq and Sanger sequencing.

The study design used for the genome-wide differential RE association analysis of epilepsy was a case control analysis using a mouse model of acquired symptomatic TLE (Fig. 1A). High-throughput sequencing of mRNA (RNA-seq) was carried out in whole hippocampus samples from 100 epileptic and 100 control littermate mice (same age) as previously described (Johnson et al. 2015a). All mice underwent continuous video monitoring for 14 consecutive days beginning 28 days following pilocarpine-induced status epilepticus to document the occurrence and frequency of spontaneous behavioral epileptic seizures, as previously described (Mazzuferi et al. 2012). All RNA-seq profiles generated in epileptic and control mice (*n* = 200) were initially used for the prediction of RNA-editing events, and the identified RE events were then further analyzed in epileptic and control mice to identify differential RE (DRE) between cases and controls (Methods).

### Predicting RNA-edited sites in the mouse hippocampus

To maximize the likelihood of identifying true RE events, we carried out genome-wide prediction of RE events using RNA sequencing data from all 200 mice hippocampi. After removal of known single nucleotide variants (SNVs) and using stringent filters for base (quality score > 25) and mapping quality (quality score > 20) (see Methods), we identified 201,322 unique mRNA sites with an alternate allele significantly different from its corresponding DNA sequence in at least one sample (BH-corrected *P-*value < 0.05, using REDItools) (Supplemental Fig. S1; Picardi and Pesole 2013). This set of candidate RE sites was further analyzed to minimize other potential sources of systematic bias, including variant distance bias (VDB; i.e., to account for false variants that tend to occur at a fixed distance from the end of reads), removal of read sequences with very high similarity (>95%) with other genomic regions, and strand bias (i.e., to account for variants observed only in either the forward or reverse strand of mapped reads) (see Methods for details). Next, we filtered out sites that resided within mouse regions harboring genome duplication events and the sites within 4 bp of an exon-intron boundary (the latter because RNA-seq mapping near exon boundaries tends to be unreliable) (Danecek et al. 2012). Finally, we required the RNA-edited sites to be detected in at least 5% of all samples. After these quality control and filtering steps, we identified a set of 9277 sites predicted to be RNA-edited in the mouse hippocampus (Supplemental Fig. S1) and for which there were no systematic differences in the mapping, base quality, and read coverage between epileptic and control hippocampi (Supplemental Fig. S2).

Since RE is an enzyme-catalyzed event and the responsible enzymes tend to edit nucleotides in a cluster, especially in the 3′ UTR or introns of the gene (Park et al. 2012), we reasoned that, potentially, an isolated RNA-edited site is more likely to be a false-positive prediction than two or more RNA-edited sites that occur in close proximity to each other (i.e., that are clustered together) (Danecek et al. 2012). Therefore, we took the additional filtering step of generating a refined list of RE sites which excluded all sites not residing within 50 bp of another RE site (Danecek et al. 2012). Considering only sites clustered on the same strand and consisting of the same base substitution, this resulted in a set of 4780 sites that we refer to as “clustered” RNA-edited sites. In the subsequent analyses, we considered both the set of 4780 “clustered RNA-edited” sites as well as the full set of 9277 RE sites (termed “all” RNA-edited sites). RNA-seq reads corresponding to the full set of RNA-edited sites identified in this study are publicly available at Figshare.com and can be accessed using this link: https://dx.doi.org/10.6084/m9.figshare.4476323.

### Differential RNA-editing analysis in epilepsy

We then set out to identify, at the genome-wide scale, RNA-edited sites associated with epilepsy by testing if the degree of RE at each site is significantly different between epileptic case and healthy control hippocampus samples. To this aim, we employed generalized linear mixed models (GLMMs) using a binomial distribution and the nonparametric Wilcoxon signed-rank (WSR) test to identify RNA-edited sites that are significantly differentially edited between cases and controls. Since parametric and nonparametric approaches have their respective advantages and disadvantages, to identify differential RE sites here, we took the conservative route of taking the overlap of the results obtained by both approaches (see Methods). Using this strategy, considering the set of 4780 clustered RE sites, we identified 134 RE sites (in 39 unique protein coding genes) as differentially edited (differentially RNA-edited, also DRE) between epileptic and control hippocampi (Monte Carlo *P*-value < 1.0 × 10^−3^). Similar analysis of the 9277 nonclustered RE sites (i.e., “all” RE sites), identified 256 DRE sites (in 87 unique genes; Monte Carlo *P*-value < 1.0 × 10^−3^) (Fig. 1B; Supplemental Figs. S3, S4A; Supplemental Tables S1, S2). Overall, A-to-I DRE was observed in 56 genes for all DRE and 31 for the clustered set, while C-to-U editing was observed in 31 and 10 genes for all and clustered DRE sites, respectively (Fig. 1B). For the all RE set, the ratio between the number of DRE sites per gene with C-to-U DRE is 3.9 (6.8 for the clustered DRE), while for A-to-I, it is 2.1 (1.9 for the clustered DRE) (Fig. 1B). This is consistent with a previous observation that C-to-U editing tends to occur in clusters (Rosenberg et al. 2011; Park et al. 2012).

The majority of DRE sites between epileptic and healthy hippocampus (95.5% for clustered sites and 92.2% for all sites) belonged to the two most prevalent known classes of editing (i.e., A-to-I and C-to-U) (Fig. 1B; Supplemental Fig. S4B; Danecek et al. 2012; Park et al. 2012). Thus, in the set of 134 clustered DRE sites, we found that 79.5% of the protein coding genes were A-to-I edited (Supplemental Table S2; Supplemental Fig. S3). Additionally, we identified 10 transcripts with 68 C-to-U sites DRE between epileptic and control mouse hippocampus, which also included four C-to-U sites which had been previously reported as RNA-editing events in macrophages (Supplemental Table S2; Hassan et al. 2014).

Among the sites DRE between epilepsy cases and controls, the percentages of noncanonical sites were 7.8% and 4.5% for all and clustered data sets, respectively. Noncanonical sites are often considered to be an indication of a potential false-positive rate of detection of RE events (Danecek et al. 2012). In our analysis, the percentage of noncanonical sites prior to the analysis of differential RE was 22% and 15% for all and clustered sites, respectively. To assess the potential effect of noncanonical RE sites on the accuracy of our DRE analysis, we therefore carried out a simulation study where RNA-editing percentages were increased or decreased at random (termed “noise”) under two different statistical frameworks (Supplemental Text S1). We found that when ∼20%–25% of the sites are “noise,” the estimate of the false-positive rate for DRE is ∼10% for all RE sites and ∼5% for “clustered” RE sites, respectively.

The difference between mean editing levels for sites found to be significantly DRE between epileptic and control hippocampus ranged between −15% and 15%, with the majority (72%) of the sites having small RE differences (<5%) (Supplemental Table S2). In addition, we observed that 75% of the DRE sites have higher mean RE levels in epileptic hippocampi compared to controls (Fig. 1C). Critically, the identified DRE sites were not enriched for genes that were differentially expressed (false discovery rate [FDR] < 5%) between epileptic and control hippocampi for either all DRE sites (Fisher's exact test [FET] *P*-value = 0.57) or clustered DRE sites (FET *P*-value = 0.50). However, we found that DRE sites were significantly enriched in highly expressed genes (based on average fragments per kilobase per million [FPKM] values from all 200 mice; Gene Set Enrichment Analysis [GSEA], FDR < 0.001 for both all and clustered DRE sites).

### Validation of differentially RNA-edited sites

Because prediction of RNA-DNA mismatches is prone to multiple sources of error (Kleinman and Majewski 2012; Lin et al. 2012; Pickrell et al. 2012), we sought to further corroborate the identified differentially RNA-edited sites. First, we investigated whether the sites that were DRE between epileptic and control mouse hippocampus have been previously independently reported as RNA-edited sites, based on the two publicly available data sets of RE in the mouse—DARNED (Kiran and Baranov 2010) and RADAR (Ramaswami and Li 2014; see Methods). We observed that 22% and 25% of clustered and all DRE sites, respectively, have been previously reported as sites of RE in normal tissues (Fig. 1B). This overlap between DRE sites and previously reported RNA-edited sites was tested using the hypergeometric test and GSEA, which showed a significant enrichment of known (previously reported) RE sites within our set of DRE sites (hypergeometric *P*-value = 1.6 × 10^−8^; GSEA *P*-value < 1.0 × 10^−5^) (Supplemental Fig. S2D). This significant overrepresentation of previously reported RE sites provides an independent line of evidence supporting the validity of the predicted RE sites. Second, we undertook Sanger sequencing in 22 RE sites which consisted of 14 clustered and six nonclustered sites predicted to be DRE between cases and controls, as well as two sites which were removed as false-positive predictions under our stringent filtering criteria. The predicted DRE sites consisted of fourteen A-to-I, four C-to-U, and two noncanonical RE sites. Using 10 mouse hippocampus samples (five cases and five controls), the percentage of RE was estimated from Sanger sequencing using ab1 Peak Reporter Tool (Roy 2014). All 22 sites were validated as either true DRE sites, including the previously unreported (for brain tissue) C-to-U edited site, or true negatives. Overall, the percentage of RE estimated from Sanger sequencing agreed with estimates from our RNA-seq analysis—*R*^2^ = 0.75 and *P*-value < 2.2 × 10^−16^ (Fig. 1D). Sanger sequencing also validated the five DRE sites not previously reported in the DARNED or RADAR data sets. These analyses establish a good level of agreement between the predicted and Sanger-validated DRE sites. We therefore explored the potential functional consequences of the transcripts that showed DRE between epilepsy cases and controls.

### Annotation and functional enrichment analysis of genes DRE in epilepsy

**Figure 2.**
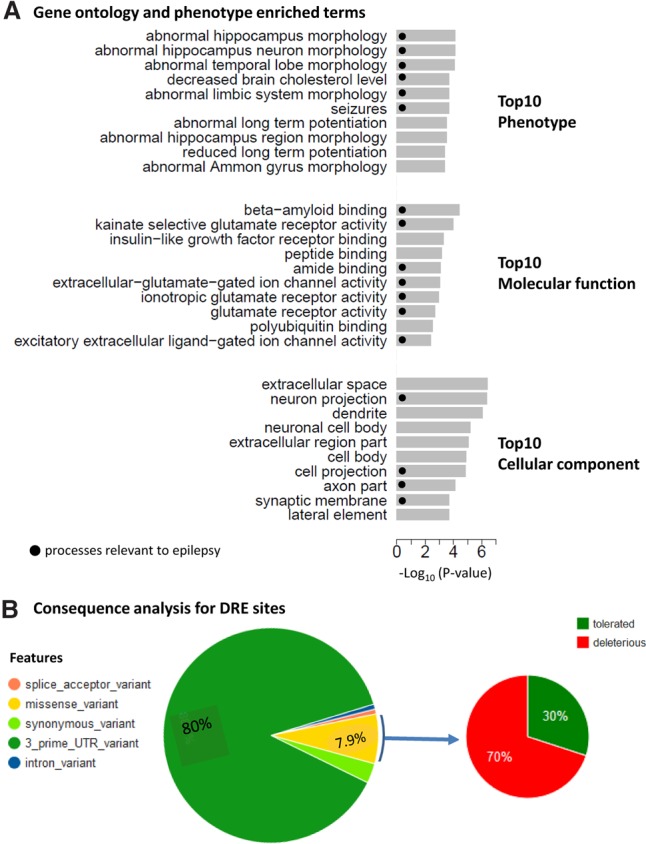
(*A*) Functional annotation of genes harboring differential RNA-edited clustered sites for Gene Ontology (GO) categories phenotype, molecular function, and cellular component. (*B*) Consequence analysis of the differential RNA-edited clustered sites.

To investigate whether genes DRE between epilepsy cases and controls are involved in functional processes and pathways relevant to epilepsy, we carried out functional enrichment analysis taking the conservative approach of using the set of genes that are expressed in mouse hippocampus as the background (see Methods). We observed significant enrichment for processes with Gene Ontology (GO) or phenotype terms relevant for epilepsy and neuronal excitability among the genes impacted by DRE, including “neuron projection” (BH-adjusted *P*-value = 2.4 × 10^−5^ for genes with clustered DRE and *P*-value = 4.2 × 10^−5^ for genes with all DRE, respectively), “synapse” (BH-adjusted *P*-value = 2.2 × 10^−3^ for genes with clustered DRE and *P*-value = 3.7 × 10^−3^ for genes with all DRE, respectively), and “seizures” (BH-adjusted *P*-value = 1.4 × 10^−2^ for genes with clustered DRE and *P*-value = 1 × 10^−2^ for genes with all DRE, respectively) (Fig. 2A; Supplemental Fig. S4C).

To further validate our findings, we also considered RE sites that had been previously reported with high confidence in the DARNED and RADAR databases (Kiran and Baranov 2010; Ramaswami and Li 2014). Union of these two databases resulted in 8891 sites previously reported to be RNA-edited in mice, and of these, 1652 sites had fair coverage, i.e., base and mapping quality score > 20 and covered by at least 10 reads in 5% (*n* = 10) of samples (Supplemental Fig. S5A). Next, we tested whether these previously reported RE sites were differentially edited between epileptic and control hippocampi (as previously described), resulting in the identification of 114 DRE sites (in 70 genes) (Supplemental Fig. S5A; Supplemental Table S3). The majority of these DRE sites resided in the intronic and/or exonic regions of the genes (Supplemental Table S3). In addition to the expected A-to-I transitions, we also observed five DRE sites with C-to-U editing. The 70 genes impacted by DRE in epileptic mouse hippocampus had significant enrichment for GO term “kainate selective glutamate receptor activity” (BH *P*-value = 0.04) (Supplemental Fig. S5B). The two databases DARNED and RADAR were built from the union of RE sites identified across heterogeneous tissue types including brain. The identification of functionally related GO terms from DRE analysis of RE sites previously reported in DARNED and RADAR and the set of RE events identified in this study provide an independent level of evidence supporting our predictions and suggests we would have missed many DRE sites potentially relevant to epilepsy had we restricted our analysis to previously known sites.

To further investigate whether the observed functional overrepresentation in transcripts impacted by DRE (Fig. 2A) is not simply a result of genes differentially expressed in epilepsy, the functional enrichment analysis was repeated for genes significantly (FDR < 0.05) differentially expressed between epileptic cases and controls (Supplemental Fig. S6A; Supplemental Table S4). Here, we observed little overlap between GO terms enriched among genes DRE in epilepsy and those enriched among genes differentially expressed in epilepsy, consistent with our earlier observation that DRE sites are not enriched for genes differentially expressed in epilepsy (above). These results suggest that differential gene expression alone does not account for the observed differences in DRE between epilepsy cases and controls. While some functional terms enriched among genes highly expressed in hippocampi overlapped with those enriched among genes DRE in epilepsy (e.g., “neuron_projection” and “synapse”), these terms were very similar for both epileptic cases and nonepileptic controls (Supplemental Fig. S6B).

### Consequence analysis of DRE sites

We used the variant effect predictor from Ensembl to characterize the effects of edited sites on the function of genes affected by DRE (Fig. 2B; Supplemental Table S5). Consistent with previous observations (Blanc and Davidson 2011; Danecek et al. 2012; Gu et al. 2012; Blanc et al. 2014), we observed that the majority (all = 77.3% and clustered = 80%) of the DRE sites resided in the 3′ UTR of the gene (Liu et al. 2014), while 21.9% of all DRE sites and 11.1% of the clustered DRE sites resided in the protein coding regions of the gene (Fig. 2B). SIFT (Single submission returns functional predictions) analysis predicted 66.7% of all and 70% of clustered exonic DRE sites to be deleterious (Fig. 2B; Supplemental Fig. S4D; Supplemental Tables S5, S6).

### Cell-type enrichment and motif analysis

To provide further insights into the cellular origin of each RE type (A-to-I and C-to-U), we used a published set of cell-type marker genes obtained by single-cell RNA-seq analysis of mouse hippocampus (Zeisel et al. 2015; see Methods). The marker genes were used to test for cell-type specificity in the set of genes DRE in epilepsy (Supplemental Fig. S7A). C-to-U edited genes were most strongly enriched for microglia and oligodendrocytes, while A-to-I edited genes were enriched for astrocytes as well as interneurons. Overall, we found no enrichment for endothelial, mural, and ependymal cell types. Interestingly, A-to-I and C-to-U genes with DRE were almost mutually exclusive to each other in terms of their cell-type specificity. This is keeping with previous data showing that C-to-U editing is highly expressed and active in macrophages/microglial cell types (Rosenberg et al. 2011). To validate the identification of C-to-U editing and to corroborate the enrichment of C-to-U edited sites in microglial cell types, we downloaded a previously published data set (E-GEOD-66211) containing RNA-seq profiles for microglial cells (Cronk et al. 2015). We found that 25% of the C-to-U sites from the clustered and 18% from the all predicted sites identified in our study were conserved in microglial cells. These results provide a level of independent evidence to support the validity of our C-to-U RE predictions but suggest cell types other than microglia may also contribute to the identified C-to-U editing.

Previous studies have shown that C-to-U editing has been associated with a nucleotide motif found ±5 bp to the edited base (Rosenberg et al. 2011). We therefore extracted ±5-bp-long sequences relative to the 135 C-to-U editing sites from the mouse genome to calculate the nucleotide frequency for each position, which is represented in the form of sequence logo (Supplemental Fig. S7B). The identified motif was significantly similar (*P*-value = 0.004) (Supplemental Fig. S7B) to the previously described sequence motif shown to be associated with C-to-U edited sites (Rosenberg et al. 2011). In addition to the motif, we observed that the differential RE sites were also AT-rich, in keeping with previous observations showing that identified C-to-U editing tends to preferentially occur in AT-rich regions (Supplemental Fig. S7C; Blanc and Davidson 2011; Roberts et al. 2013; Blanc et al. 2014). These observations are consistent with previously proposed mechanisms in which APOBEC1 targets require a sequence with high AT content for efficient mRNA editing (Rosenberg et al. 2011). In addition, the mRNA levels of several *Apobec* family genes, i.e., the key enzymes for C-to-U RNA editing, were differentially expressed between epileptic and control mice hippocampus, with increased expression in the epileptic hippocampus (Supplemental Fig. S7D). Taken together, the enrichment of microglia marker genes among C-to-U DRE sites, their frequent occurrence in clusters (2.6 and 5 sites per gene for all and clustered DRE, respectively), their occurrence within AT-rich regions and the significant differential expression of genes encoding enzymes responsible for C-to-U editing suggests that the C-to-U DRE sites could potentially be catalyzed by the APOBEC family of enzymes. Similarly, ADAR and ADARB1, key enzymes for facilitating A-to-I editing, are significantly differentially expressed as well as highly expressed in both cases and controls [*Adar*: mean FPKM value = 70, log_2_ (fold change) = −0.19, *P*-value = 1 × 10^−10^; *Adarb1*: mean FPKM value = 1417, log_2_ (fold change epilepsy/control) = −0.22, *P*-value = 8 × 10^−13^]. However, this observation alone is unlikely to explain the full spectrum of observed A-to-I DRE in our study where A-to-I editing represents a mixture of high (73%) and low (27%) edited sites with respect to control hippocampus, suggesting potential involvement of cofactors.

### Relationship between DRE sites and seizures and epilepsy

**Figure 3.**
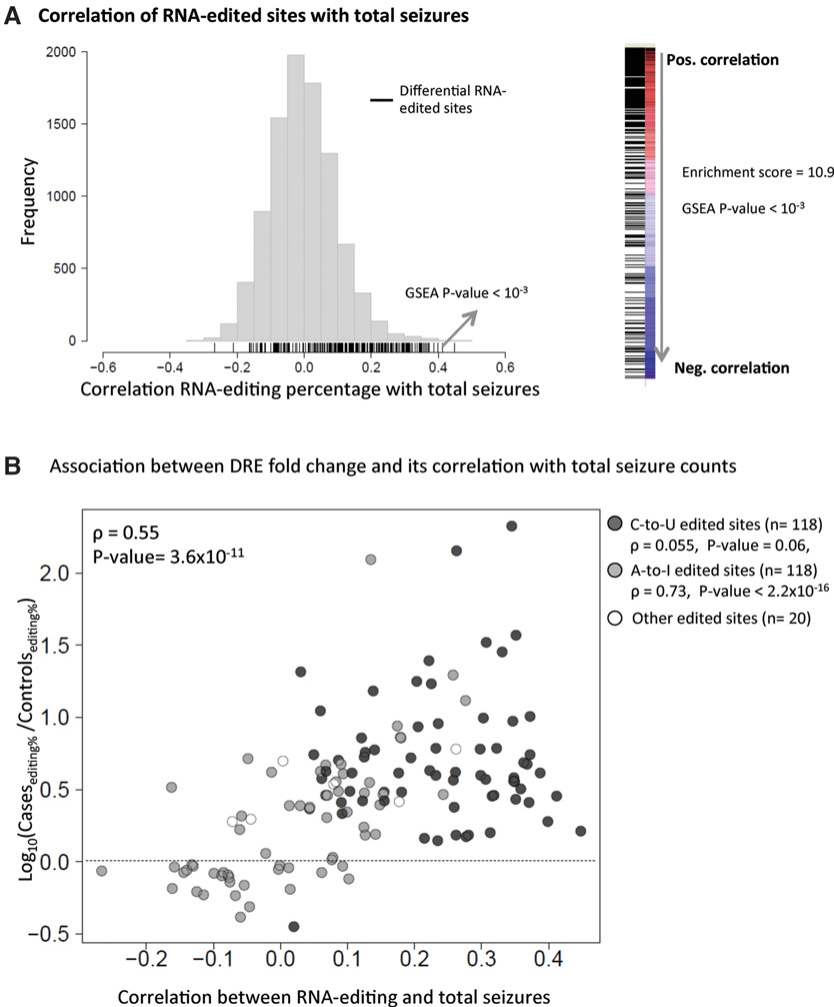
Relationship between RNA editing in the hippocampus and seizure frequency (here, measured as total seizure counts over a standardized period of 14 continuous days of monitoring). (*A*) Correlation between RNA-editing percentages and total seizure counts in the 100 epileptic mice. (*B*) Association between RNA-editing fold change and correlation between RNA-editing percentage and total seizures in epileptic mice. C-to-U sites are represented as solid black circles, while A-to-I sites are solid gray circles. All other sites are represented as white circles with a black outline.

To further investigate the role of DRE in epilepsy, we tested the association between the level of DRE and frequency of seizures observed in the epileptic mice. Here, seizure frequency (SF) was assessed by two weeks of continuous video monitoring of behavioral seizures in 100 epileptic mice (see Methods). First, for each of the RE sites identified in the mouse hippocampus (n = 9277), we calculated the (nonparametric) Spearman correlation between the RE percentage and the total number of seizures observed in each epileptic mouse. These Spearman correlations were normally distributed, with mean correlation close to zero, suggesting that the majority of edited sites are not associated with epilepsy (Fig. 3A). In contrast, when we considered the RE sites that were significantly differentially edited between cases and controls (i.e., the DRE sites), the percentage of RNA editing in cases was significantly and positively correlated with the number of seizures (GSEA FDR < 10^−3^ for both clustered and all DRE sites) (Fig. 3A). We also found that the higher the fold change in RE percentages (between epileptic and control mice), the stronger is the association between RE and total number of seizures (r = 0.55, P < 2.2 × 10^−11^ for clustered DRE; r = 0.51, P < 2.2 × 10^−16^ for all DRE) (Figure 3B). Interestingly, only sites with A-to-I editing showed a significant correlation with seizure frequency (ρ = 0.73, *P*-value <2.2 × 10^−16^) (Fig. 3B), while C-to-U edited sites were mostly up-regulated but not strongly correlated with seizures (ρ = 0.055, *P* = 0.06).

**Figure 4.**
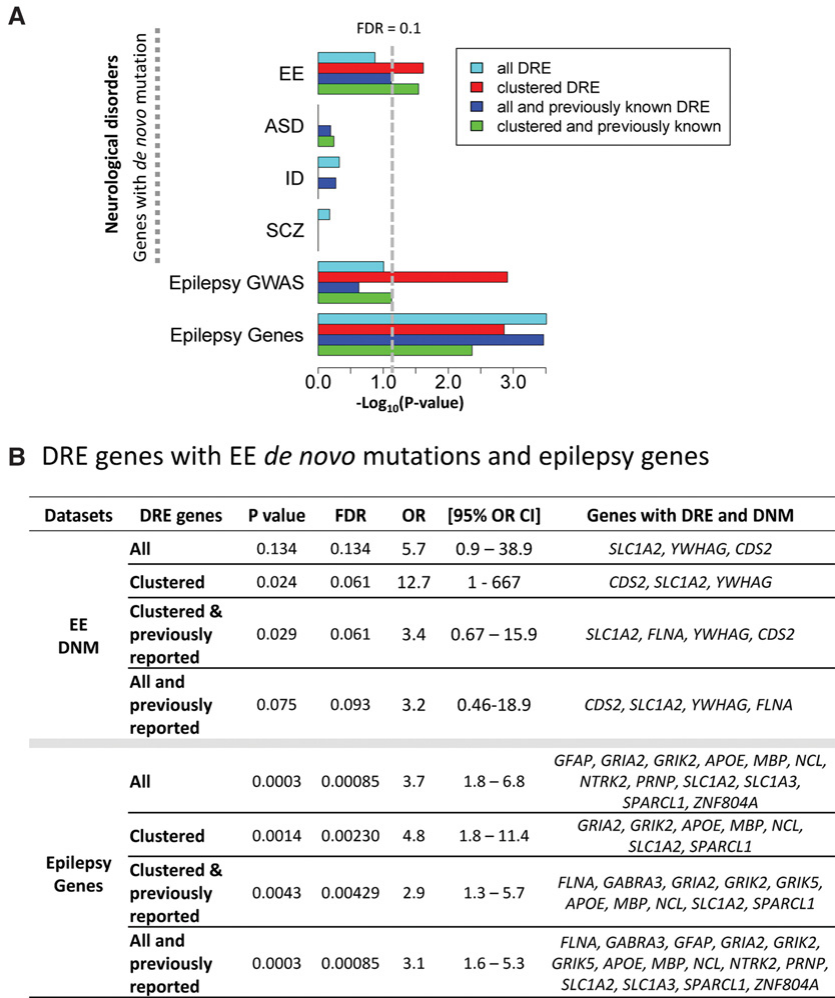
(*A*) For genes impacted by differential RNA editing, enrichment for de novo mutations from patients with epileptic encephalopathy (EE), autism spectrum disorder (ASD), intellectual disability (ID), and schizophrenia (SCZ); enrichment of association to genetic generalized epilepsy (“Epilepsy GWAS”) and enrichment for known epilepsy genes (Epilepsy Genes) (as defined by DisGeNET). (*B*) Table summarizes the enrichment *P*-value, odds ratio (OR), and 95th percentile of the confidence interval of the OR and lists the genes contributing to the enrichment of EE de novo mutations (*top*) and known epilepsy genes (*bottom*) among genes differentially RNA-edited in epilepsy.

We also investigated whether the set of genes with DRE between epileptic and control mice was enriched for genes associated with epilepsy when mutated, and to assess specificity, we also tested for enrichment for genes for other neuropsychiatric diseases. To this end, we tested the set of genes DRE between epileptic and control hippocampi for enrichment of validated nonpolymorphic de novo single nucleotide variant mutations (DNMs) identified in neurodevelopmental whole-exome sequencing (WES) studies that shared similar sequencing technologies, coverage criteria, and variant calling methodology (Johnson et al. 2015b). Collectively, the neurodevelopmental disease cohort consisted of 5738 nonoverlapping published parent-offspring trios across four disease phenotypes; epileptic encephalopathy (EE, *n* = 356), autism spectrum disorder (ASD, *n* = 4186), schizophrenia (SCZ, *n* = 1004), and intellectual disability (ID, *n* = 192) (see Methods for cohort references). For controls, we used 1891 nonneurological control samples as previously reported (Johnson et al. 2015b). The genetic relationship of DRE genes to epilepsy was tested using a FET (two-tailed) to empirically compare the rates of DNMs overlapping the consensus CDS of DRE genes in case and control cohorts. We considered DRE sites in three groups: (1) clustered, (2) all sites significantly DRE, and (3) from among all sites significantly DRE, the subset consisting of those previously reported as being RNA-edited from the DARNED and RADAR data sets. Considering nonsynonymous DNM (nsDNM) consisting of all missense, nonsense, and splice-site mutations, we found evidence that the human orthologs of the mouse genes DRE in epilepsy were enriched for genes that, when mutated, are associated with epileptic encephalopathy (Fig. 4A,B). No enrichment was detected for other neuropsychiatric disease genes. These results suggest that the DRE events associated with symptomatic acquired epilepsy in the mouse overlap with genes that, when mutated, confer genetic risk for epilepsy. To investigate this possibility further, we tested genes DRE in the mouse model of acquired epilepsy for enrichment of association to common forms of epilepsy using published epilepsy genome-wide association study (GWAS) data (International League Against Epilepsy Consortium on Complex Epilepsies 2014; see Methods). Here, we observed that clustered DRE genes were significantly (*P*-value = 0.001) enriched for association to generalized epilepsy (Fig. 4A,B). We also tested DRE genes for enrichment of known epilepsy genes as defined by the DisGeNET database (Bauer-Mehren et al. 2010; Pinero et al. 2015; Queralt-Rosinach et al. 2016) and observed significant enrichment of known epilepsy genes among both all and clustered DRE genes (Fig. 4A,B). These results are consistent with the set of genes DRE in acquired epilepsy being enriched for genes that have a genetic relationship to epilepsy.

### Analysis of mouse genes with differential RNA editing in human epileptic hippocampus

To explore the hypothesis that the DRE identified in the epileptic mouse hippocampus might be relevant to human epilepsy, we analyzed RNA sequencing data from dentate gyrus samples ascertained from six patients with mesial temporal lobe epilepsy together with matched whole-exome sequence data (Griffin et al. 2016). Considering the set of genes DRE in the mouse epileptic hippocampus, 21 (18.4%) of all and 11 (28%) of clustered DRE genes were also found to be RNA-edited in the human epileptic hippocampus (Supplemental Fig. S8; Supplemental Table S7). These results highlight that a subset of genes DRE in the mouse epileptic hippocampus are also RNA-edited in the human epileptic hippocampus, but further analysis will be required to determine if DRE in these genes is associated with human epilepsy.

## Discussion

Recent intense scrutiny of RNA editing has led to a better appreciation of the potential sources of bias and error associated with the identification of RE (Kleinman and Majewski 2012; Lin et al. 2012; Pickrell et al. 2012). Consequently, improved methods to robustly characterize genome-wide RE events (Danecek et al. 2012; Ramaswami et al. 2012; Picardi and Pesole 2013) have enabled genome-wide characterization of RE in several, mostly nondiseased, tissues (Danecek et al. 2012; Park et al. 2012; Lagarrigue et al. 2013; Bazak et al. 2014; Blanc et al. 2014; Han et al. 2015). To date, however, only a few studies have reported RE events at a genome-wide scale in disease tissues; for example, Han et al. (2015) showed that in various cancer types RE events are associated with survival and drug resistance. A role for RE in diseases such as epilepsy has been suggested, but these findings have been limited to candidate gene studies (Feldmeyer et al. 1999; Balik et al. 2013), akin to the candidate gene association studies undertaken prior to genome-wide association studies. Here, we undertook a genome-wide differential RE association study of epilepsy.

In this study, we present a comprehensive framework for testing differential RNA-editing events in disease. We aimed to detect RNA-edited sites in an accurate manner and in an adequately powered sample set, and therefore we started from the analysis of a large sample of 200 mouse hippocampi. We then used a stringent data filtering strategy that included detection of the edited site in at least 10 samples and removal of all known single nucleotide variants and indels, estimated to constitute >95% of SNPs in outbred mice and with novel variant rate of 0.8% in the NMRI mice strain (used in this study) (Yalcin et al. 2010). We further validated a subset of predicted differentially RNA-edited sites using Sanger sequencing and repeated our analyses considering only previously reported RNA-edited sites, which supported the validity of the identified DRE.

Our genome-wide DRE analysis yielded a set of 256 sites (in 87 unique genes) significantly differentially edited between epileptic and control hippocampi, of which a subset of 134 sites (in 39 unique genes) were clustered (i.e., had at least one other RE with the same nucleotide transition within 50 bp). Genes with DRE (both all and clustered subsets) were specifically enriched for functional terms highly relevant to neural processes and epilepsy. Notably, 70% of exonic DRE sites are predicted to be deleterious. These results suggested a potential relationship between DRE and the occurrence of seizures and epilepsy. To investigate the potential role of transcripts impacted by DRE in epilepsy, we tested whether transcripts significantly differentially edited in the mouse model of epilepsy were enriched for genes with a genetic relationship to epilepsy. Considering both all and clustered DRE, we found genes impacted by DRE are enriched for genes impacted by de novo mutations in patients with epileptic encephalopathy, as well as significantly enriched for association to common forms of generalized epilepsy. These enrichments suggest that RE events in the mouse epileptic hippocampus are nonrandomly occurring and are potentially disrupting important genes that modulate specific pathways and processes relevant for human epilepsy. In keeping with this, we found that RNA editing is also associated with seizure frequency in epileptic mice and that fold change of DRE was significantly and positively correlated with seizures. Given the high expression of the genes with differential RE editing and their known functional relevance to epilepsy, it is possible that even a relatively small change in RNA editing might have a biological role, in particular when multiple genes with differential editing are impacting the same pathways and biological processes in the hippocampus.

In summary, our genome-wide analysis of DRE in epilepsy identified hundreds of DRE sites which are enriched for genes associated with epilepsy and which are significantly correlated with seizures in the mouse epilepsy model. Analysis of the cell types and molecular processes associated with the epilepsy-associated RNA editing reveals cell-type–specific RNA editing and a potential involvement of both microglial and neuronal processes (Chugh et al. 2013; Zhan et al. 2014). These results highlight a potential role for RNA editing in epilepsy. Our results prompt further research to determine the genome-wide role of DRE in acquired human epilepsy.

## Methods

### Mouse pilocarpine model of epilepsy

Status epilepticus was induced in male NMRI mice (weighting 28–32 g at the beginning of the study) by a single injection of pilocarpine as previously described (Mazzuferi et al. 2012). Briefly, animals were injected intra-peritoneally (i.p.) with 1 mg/kg of N-methylscopolamine bromide 30 min prior to pilocarpine treatment (300 mg/kg; i.p.). Within 10 to 45 min after pilocarpine treatment, animals displayed generalized clonic-tonic seizures that progressed to continuous convulsive activity, i.e., status epilepticus (SE). The SE lasted 3 h and was interrupted by i.p. injection of diazepam (10 mg/kg) to limit the extent of brain damage. The mice surviving SE typically show spontaneous recurrent seizures within a few days and continue to display them for several weeks (Mazzuferi et al. 2012). The seizure monitoring was performed for two weeks with a proprietary system (UCB Pharma) using simultaneous recording of locomotor activity with 3D accelerometer and video cameras. This system allows automated detection of behavioral seizures by analysis of the accelerometer signal. All behavioral seizures identified by the detection algorithm were then scored by experienced technical personnel during careful review of corresponding video clips. Approval for the research was obtained from the Imperial College Research Ethics Committee (ref: ICREC_14_2_11).

### Sample preparation for RNA-seq analysis

Total RNA was extracted from the left hippocampus of a Crl:NMRI(Han)-FR outbred mouse colony (*n* = 200; 100 controls and 100 pilocarpine-treated mice). Sample preparation for RNA sequencing (RNA-seq) was performed according to the protocols recommended by the manufacturers (TruSeq RNA kit, Illumina). Sequencing was done using an Illumina HiSeq 2000 sequencer, with paired-end 75-bp nucleotide reads according to the protocol recommended by the vendor. Raw reads were mapped to the reference mouse genome (mm10) using TopHat version 2.0.8 (Kim et al. 2013).

### Identification of RNA-edited sites

#### Previously described RNA-edited sites

Known RNA-edited sites were curated using publicly available databases: (1) Rigorously Annotated Database of A-to-I RNA editing (RADAR) (Ramaswami and Li 2014); and (2) a DAtabase of RNA EDiting (DARNED) (Kiran and Baranov 2010). DARNED also included the results of a recent study where RNA-editing sites were identified from whole brains of 15 inbred lab mouse strains (Danecek et al. 2012). These nucleotide coordinates were then used to extract reads from the samples using the “knownsites.py” program from REDItools (Picardi and Pesole 2013). We filtered coordinates that were covered by less than 10 reads in any 10 samples (irrespective of being cases or controls). In addition, the nonreference states or edited nucleotide should be covered with at least five reads.

#### Predicted RNA edited sites

Computationally, RNA editing is identified as a single nucleotide base change between DNA and RNA. We have identified RNA-edited sites using REDItools (Picardi and Pesole 2013), with the default setting for the majority of the parameters, except for: minimum base quality of 25, minimum mapping quality of 20 (probability that a read is aligned to multiple locations), probability of misalignment = 0.01 (i.e., 99% probability that a read is correctly aligned in the genome), and minimum read coverage per edited site to be 10. As described previously in Danecek et al. (2012), prediction of RE is more prone to these biases, hence, changing these parameters would reduce the number of falsely predicted RE events. In addition, to these parameters we have also used the Benjamini-Hochberg (BH)-corrected Fisher's exact test (FET) *P*-value < 0.05 for the overrepresentation of alternate allele in each site meeting the above-mentioned criteria. Initially, a site was considered to be edited if at least one sample was observed to have a significant enrichment of alternate allele (FET corrected *P*-value < 0.05 for each site). Identified edited sites were also filtered for all known mouse single nucleotide variations available from Ensembl mouse SNP database version 137. Additional biases such as strand and variant distance bias were removed as described in Danecek et al. (2012). VDB evaluates the likelihood of the mean pairwise distance of the variant bases in the aligned portion of the reads; it was calculated using SAMtools/BCFtools and the filter was set to 0.015 (Danecek et al. 2012). Strand bias was calculated by estimating overrepresentation of alternate alleles between the positive and negative strand, and a *P*-value > 0.05 was used to filter the sites/samples. Further, we removed read sequences with very high similarity (>95%) with other genomic regions and filtered out sites that resided within mouse regions harboring genome duplication events and the sites within 4 bp of an exon-intron boundary (the latter because RNA-seq mapping near exon boundaries tends to be unreliable [Danecek et al. 2012]).

Finally, we filtered all the sites that were supported by less than 10 samples with at least five reads supporting an alternate nucleotide (5% of the total samples), and the identified sites should not be in Hardy-Weinberg equilibrium (*P*-value > 0.05). In addition, for the clustered RE data set, we filtered RE sites that were not within 50 bp of another RE site at the transcript level.

### Differential gene expression analysis

Read counts per gene were calculated for each sample using HTSeq version 0.5.3 (http://www-huber.embl.de/users/anders/HTSeq) (Anders et al. 2015). Read counts per gene were further normalized across all the samples using a trimmed mean of M-value (TMM) approach as discussed in Robinson et al. (2010). Differential expression analysis was performed using the Bioconductor package edgeR version 3.2.4 (Robinson et al. 2010), and a cut-off of 5% false discovery rate was applied.

### Differential RNA-editing analysis

To identify differentially RNA-edited sites, we used two statistical methods in parallel and then integrated the results to synthesize the list of DRE sites identified by each method as follows. First, for each site, we build a generalized linear mixed model based on binomial distribution to model the probability of RNA editing as a function of case control status (fixed effect) and individual (random effect):

$$P_{E}=a+\beta \mathrm{CC}+\varepsilon ,$$

where P_E_ is the probability of the RNA-edited site to be differentially edited between cases (pilocarpine-treated mice) and controls (naïve mice), CC is the case control status, and ε is the random effect.

Second, we used the nonparametric Wilcoxon rank test to identify RNA-edited sites that had editing percentages that were different between cases (pilocarpine-treated mice) and controls (naïve mice). The Benjamini and Hochberg method was used to correct the *P*-values obtained from the test using R version 3.02 (R Core Team 2014).

Finally, the results of the GLMM and Wilcoxon rank test (i.e., BH-corrected *P*-values) were combined by means of the sdef (Synthesizing List of Differentially Expressed Features) (Blangiardo et al. 2010) package in R, using the “hmax” method to select the list of features in common at the 95% confidence interval. Briefly, the sdef method compares two (or more) lists of features (in this case, the *P*-values for each differential RNA-edited site) with the purpose of finding common features, in this case, differential RNA-editing sites commonly identified by the GLMM and Wilcoxon rank test. The overall significance of maximum overlap was tested using 10^5^ Monte Carlo permutations, and the significance level is reported as a Monte Carlo *P*-value.

Gene set enrichment analysis (Subramanian et al. 2007) was used to perform enrichment analysis of the known RNA-editing sites in the set of RNA-editing sites identified here in the mouse hippocampus, using 10^5^ permutations to estimate the significance of enrichment. The −log_10_ (*P*-value) of differential RNA editing was used as a metric to rank RNA-edited sites, and gene sets were defined as the set of known RNA-edited sites available from public databases: (1) RADAR (Ramaswami and Li 2014), and (2) DARNED (Kiran and Baranov 2010). DARNED also included the results of a recent study where RNA-editing sites were identified from whole brains of 15 inbred lab mouse strains (Danecek et al. 2012). In addition, Fisher's exact test was performed to test the enrichment of known RNA editing sites in our “all” predicted RNA-edited sites.

### Functional annotation analysis

If the edited site was within annotated gene boundaries (TSS and 3′ end), the gene was linked with a differential RNA-editing site. Genes enclosing RNA-edited sites were searched for enrichment in functional terms using the WEB-based GEneSeT AnaLysis Toolkit (Web Gestalt) (Wang et al. 2013), where the set of mouse hippocampus-expressed genes was used as a background (i.e., genes with more than log_2_[1] FPKM value in 5% of the samples). Enrichments obtained from Gene Ontology and KEGG pathways were considered as repositories for functional terms.

### Literature search for gene association with epilepsy

A manual literature search was performed using PubMed in order to check if genes have been previously associated with epilepsy, seizures, or neuropsychiatric disorders.

### Predicting the effect of RNA-editing variants

The Ensembl variant effect predictor (VEP) part of Ensembl tool release version-75 was used to functionally annotate the DRE sites. To predict the consequence of the DRE sites overlapping with exonic regions, VEP uses the SIFT (sorts intolerant from tolerant amino acid substitutions) method (Kumar et al. 2009) to infer whether an amino acid substitution in a protein will have any effect on the structure of the translated protein.

### Enrichment of genes previously linked with epilepsy

We tested genes with DRE for overlap with genes that were previously associated with epilepsy using the DisGeNET database (Bauer-Mehren et al. 2010; Pinero et al. 2015; Queralt-Rosinach et al. 2016). We performed Fisher's exact test by considering expressed genes as background; the *P*-values were FDR-corrected.

### Assessing enrichment in rare de novo mutations in neurodevelopmental disorder

De novo mutations reported in published neurodevelopmental trio whole-exome sequencing studies were collated: epileptic encephalopathy (EE, *n* = 356) (Allen et al. 2013; EuroEPINOMICS-RES Consortium; Epilepsy Phenome/Genome Project; Epi4K Consortium 2014), autism spectrum disorder (ASD, *n* = 4186) (De Rubeis et al. 2014; Iossifov et al. 2014; Ronemus et al. 2014), schizophrenia (SCZ, *n* = 1004) (Girard et al. 2011; Xu et al. 2012; Gulsuner et al. 2013; Fromer et al. 2014), and intellectual disability (ID, *n* = 192) (de Ligt et al. 2012; Rauch et al. 2012; Hamdan et al. 2014). For controls, we used 1891 nonneurological control samples from seven published studies (Iossifov et al. 2012, 2014; O'Roak et al. 2012; Rauch et al. 2012; Sanders et al. 2012; Xu et al. 2012; Gulsuner et al. 2013).

To integrate these data across their variable sources and coverage, we assumed each gene has 100% of its consensus CDS covered across all trios, as previously described (Johnson et al. 2015a). For each disorder, we included single nucleotide variant (nonsynonymous) DNMs considering all missense, nonsense, and splice-site SNV mutations. We adopted a Fisher's exact test (two-tailed) to empirically compare the rates of genes with nonsynonymous DNMs overlapping the list of DRE genes in case and control cohorts.

### Assessing enrichment in GWAS signals for generalized epilepsy

To test for enrichment of genetic association in a DRE gene set, we used versatile gene-based association study (VEGAS2) (Mishra and Macgregor 2015) to generate a gene-based association statistic (*P*-value) controlled for the number of SNPs in each gene and the LD between those SNPs. In all analyses, gene-based *P*-values were calculated using VEGAS2 and the top 10% option with 100,000 iterations and a gene window consisting of the transcriptional start and stop position of each gene. For the International League Against Epilepsy (ILAE) Consortium on Complex Epilepsies (International League Against Epilepsy Consortium on Complex Epilepsies 2014), the default 1000 Genomes European population was used to control for LD in the VEGAS2 analysis. The GWAS-enrichment statistic was calculated for the tested DRE gene set from the gene-based association *P*-values (from VEGAS2) using the Z-test-based bootstrapping method (Nam et al. 2010) (one-sided) where, for each DRE gene set, 100,000 random gene sets of the same size as the tested DRE gene set were sampled from the list of all expressed genes. We tested enrichment of DRE gene sets for association to genetic generalized epilepsy.

### Association between differential RNA editing and epileptic seizures

The nonparametric Spearman correlation was calculated between RNA-editing percentages and the total number of seizures, which were assessed as described in Mazzuferi et al. (2012). Correlations were calculated genome-wide between all RNA-edited sites (9K and 4K) and the total numbers of seizures, and the resulting distribution was plotted. To test whether DRE sites are more likely to be associated with the total number of seizures, we used GSEA. The test was performed by ranking all (36K and 16K) RNA-edited sites according to the Spearman correlation coefficients, while significant DRE sites were used as an RE list in the form of gene sets. Empirical *P*-values in the GSEA analysis were assessed by 10^5^ permutations.

### Validation of DRE sites

DRE sites were validated using the Sanger sequencing technique to confirm that RNA editing events detected were not due to polymorphism at the DNA level but were indeed RNA-editing events for 20 of our differential RNA editing events (14 clustered and seven nonclustered sites) in 10 samples. The RNA-editing percentage varied from 5% to 90% (Fig. 1D). The percentage of RNA editing was estimated from Sanger sequencing using the ab1 Peak Reporter Tool (Roy 2014).

### Conservation of DRE sites in human epileptic hippocampi

To show the conservation in human epilepsy, we have extracted reads from the combined list of DRE sites from all three approaches. RNA-seq reads were extracted from the hippocampi of six epileptic patients (Griffin et al. 2016). RNA-seq profiles from the six epileptic hippocampi were generated in two technical replicates and a RNA-site was considered to be significant if, after filtering for the technical biases as described before, they were present in at least two technical replicates.

## Data access

The RNA-seq data generated in this study have been submitted to the European Nucleotide Archive (ENA; http://www.ebi.ac.uk/ena) under accession number PRJEB18790. The catalog of identified RNA-edited sites are included in Supplemental Table S8 and their corresponding RNA-seq reads are publicly available on Figshare.com and can be accessed from https://dx.doi.org/10.6084/m9.figshare.4476323 or from Imperial College London's secure server (http://goo.gl/HoOEuT). Sanger sequencing traces have been submitted to the NCBI Trace Archive (https://trace.ncbi.nlm.nih.gov/Traces/trace.cgi) under TI numbers 2344112081-2344112279.

## Supplementary Material

Supplemental Material
