# Supplementary material for: Genome-wide analysis of differential RNA editing in epilepsy
Source: Genome Res. 2017 Mar;27(3):440–50. doi: 10.1101/gr.210740.116 (PMC5340971; doi:10.1101/gr.210740.116)
Supplement: Supplemental Material [file supp_27_3_440__index.html]

Genome-wide analysis of differential RNA editing in epilepsy — Supplemental Material 

# Genome-wide analysis of differential RNA editing in epilepsy

## Supplemental Material

- Supplement\_Figures.pdf
- Supplement\_Table\_S1.xlsx
- Supplement\_Table\_S2.xlsx
- Supplement\_Table\_S3.xlsx
- Supplement\_Table\_S4.xlsx
- Supplement\_Table\_S5.xlsx
- Supplement\_Table\_S6.xlsx
- Supplementary\_Table\_S7.docx
- Supplementary\_Table\_S8.xls
- Supplementary\_Text.docx
