# Supplementary material for: Genome-wide analysis of differential RNA editing in epilepsy
Source: Genome Res. 2017 Mar;27(3):440–50. doi: 10.1101/gr.210740.116 (PMC5340971; doi:10.1101/gr.210740.116)
Supplement: Supplemental Material [file supp_gr.210740.116_Supplement_Figures.pdf]

Supplementary Figure 1

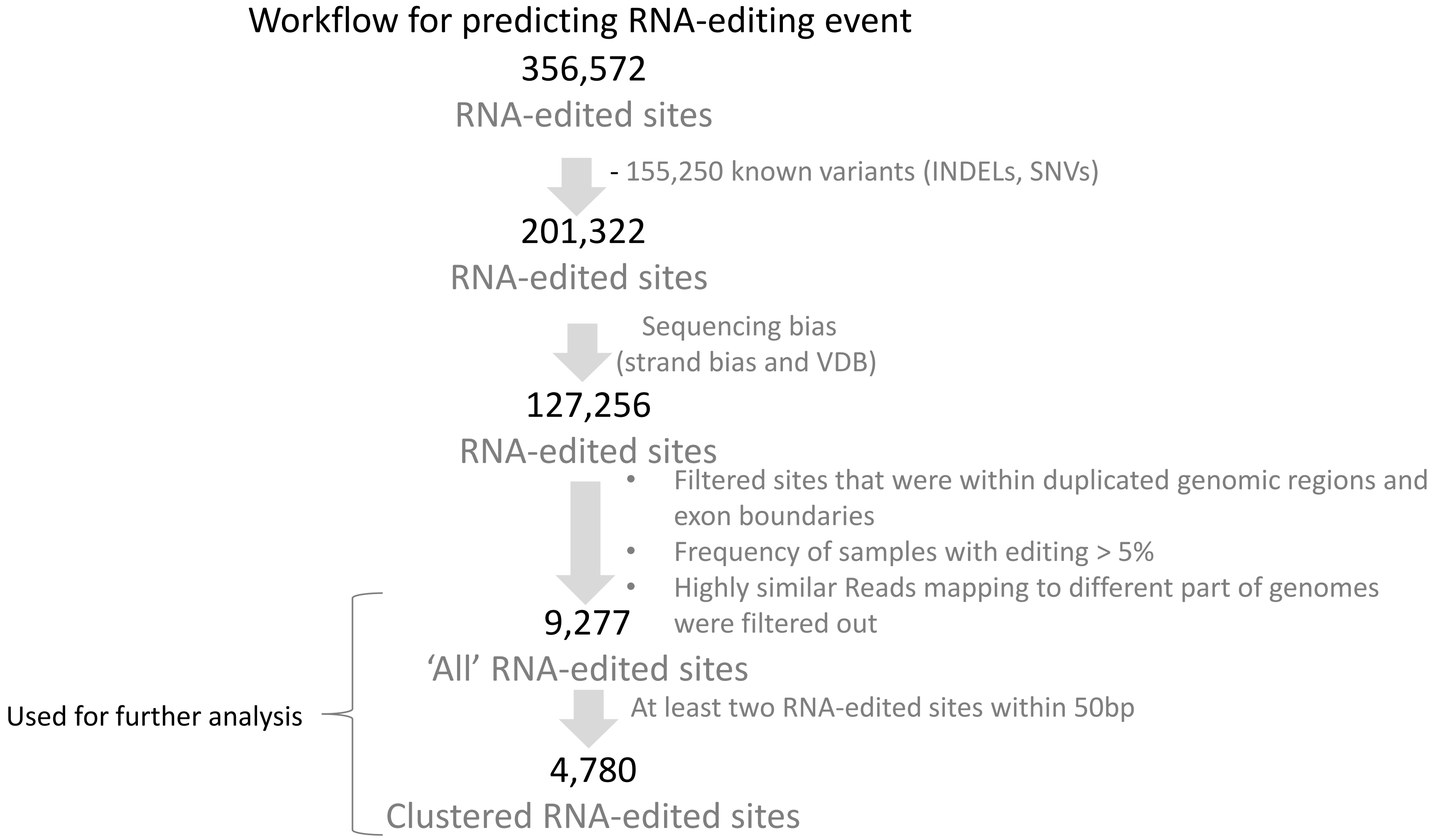

Supplementary Figure 1: Summary of the steps involved for the removal of technical artifacts from the predicted RNA-editing events: (i) removal of know variants such as SNPs and INDELs, (ii) Strand and variant distance biases (VDB), (iii) filtering for sites that lacked biological reproducibility and were near exonic boundaries and (iv) for clustered RNA-editing events we filtered sites that were not within 50 bp vicinity of another RNA editing event.

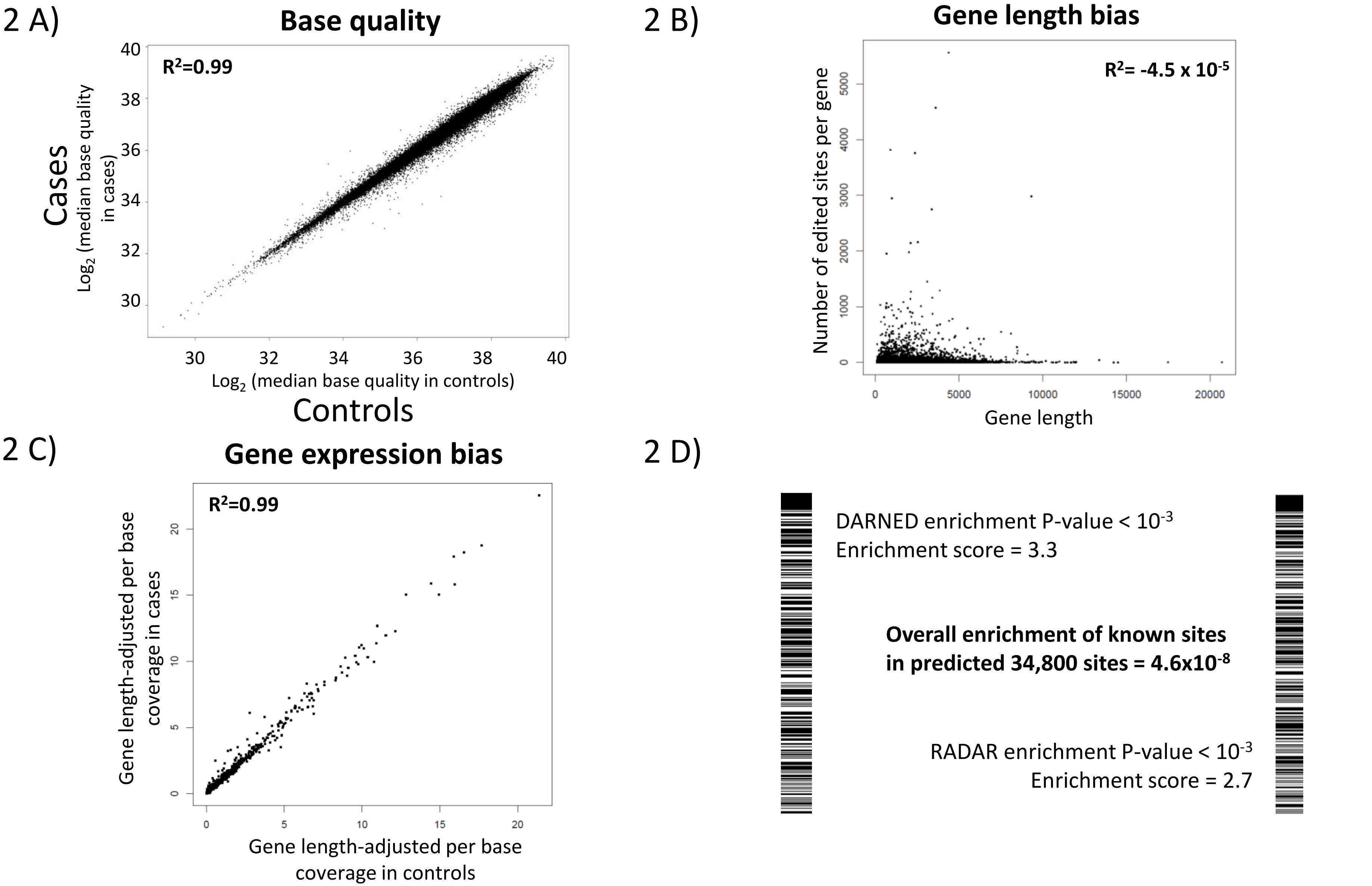

Supplementary Figure 2: To test for potential systematic biases we checked for (A) read base quality showing no differences between cases and controls, (B) low correlation between number of RNA edited sites per gene and gene length, (C) for the RNA edited sites we show equal per base read coverage for epileptic and control hippocampi, (D) GSEA analysis demonstrating enrichment of previously reported RE sites from DARNED and RADAR databases among sites DRE between epileptic cases and control.

Supplementary Figure 3

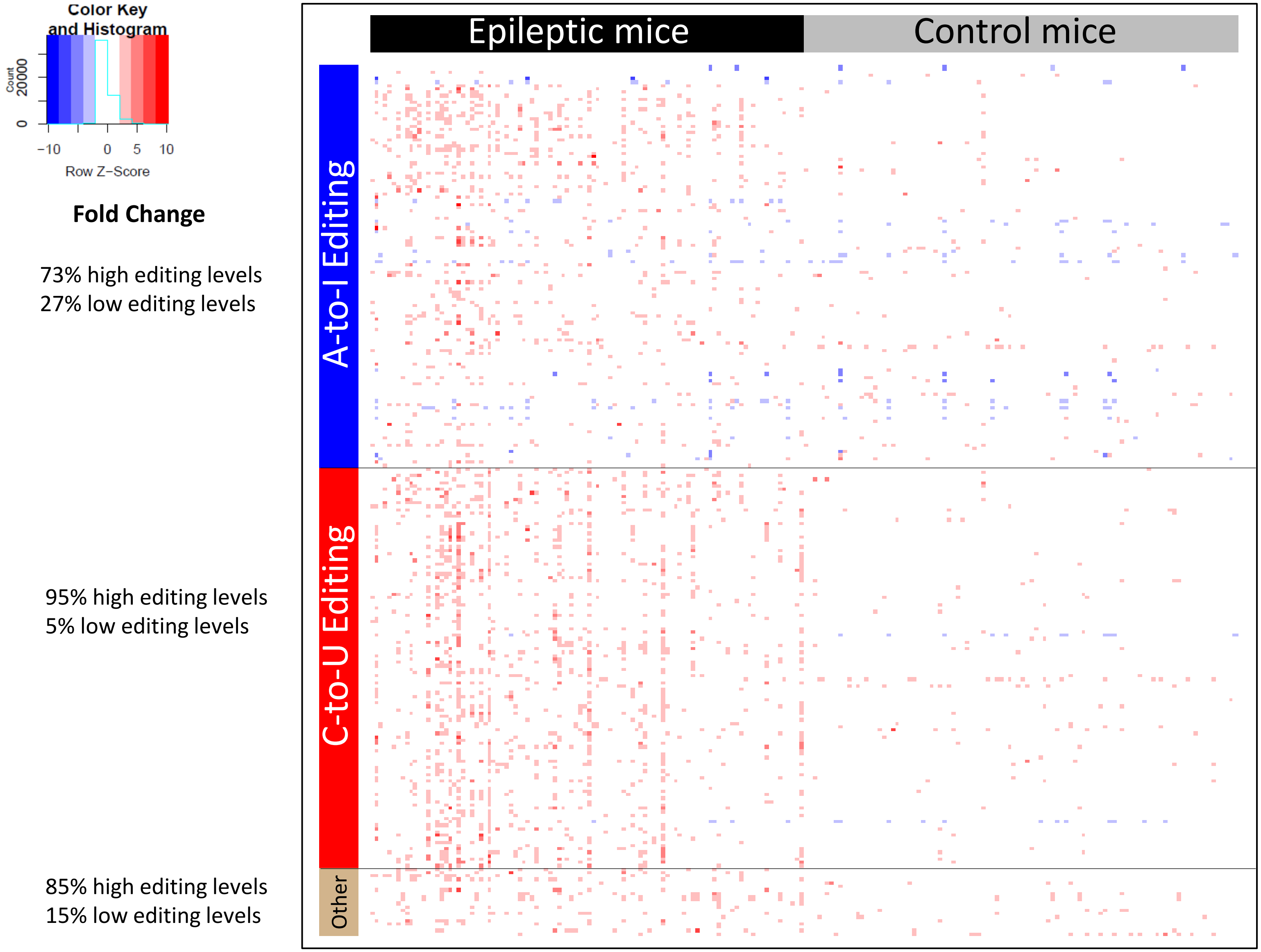

Supplementary Figure 3: Heatmap for RE editing levels for the DRE sites by type of editing. While the C-to-U sites are consistently highly edited in epileptic hippocampus (95%), the A-to-I editing represents a mixture of high (73%) and low (27%) edited sites with respect to control hippocampus.

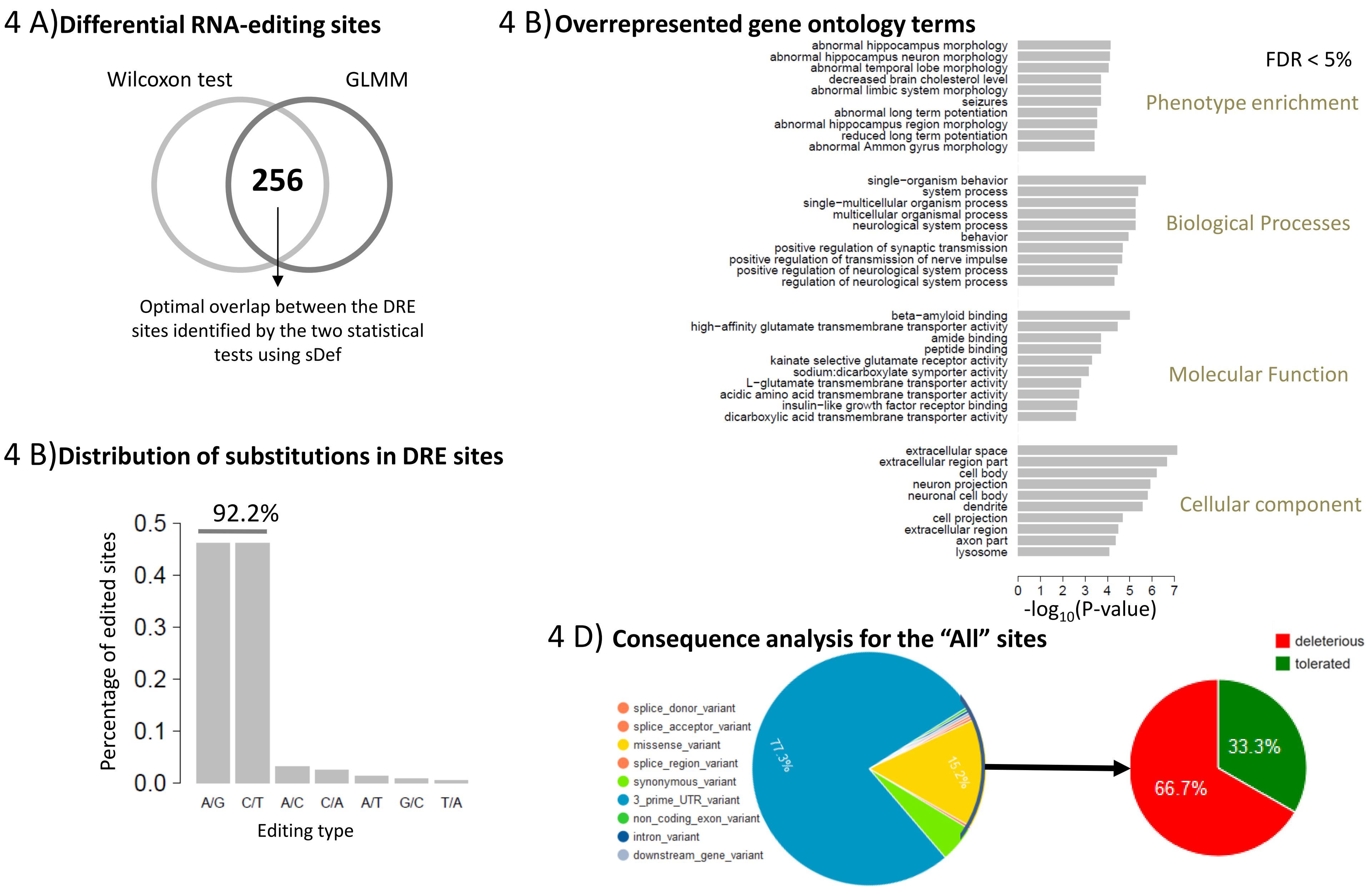

Supplementary Figure 4: Characterization of All predicted RNA-edited sites **A)** Differential RNA edited sites estimated from the two methods, **B**) Functional enrichment of the genes with differential RNA editing events by considering expressed genes as background showing enrichment for pathways highly relevant to epilepsy, **C)** Distribution of base-substitutions due to RNA editing events, and **D)** Consequence analysis for the “All” sites

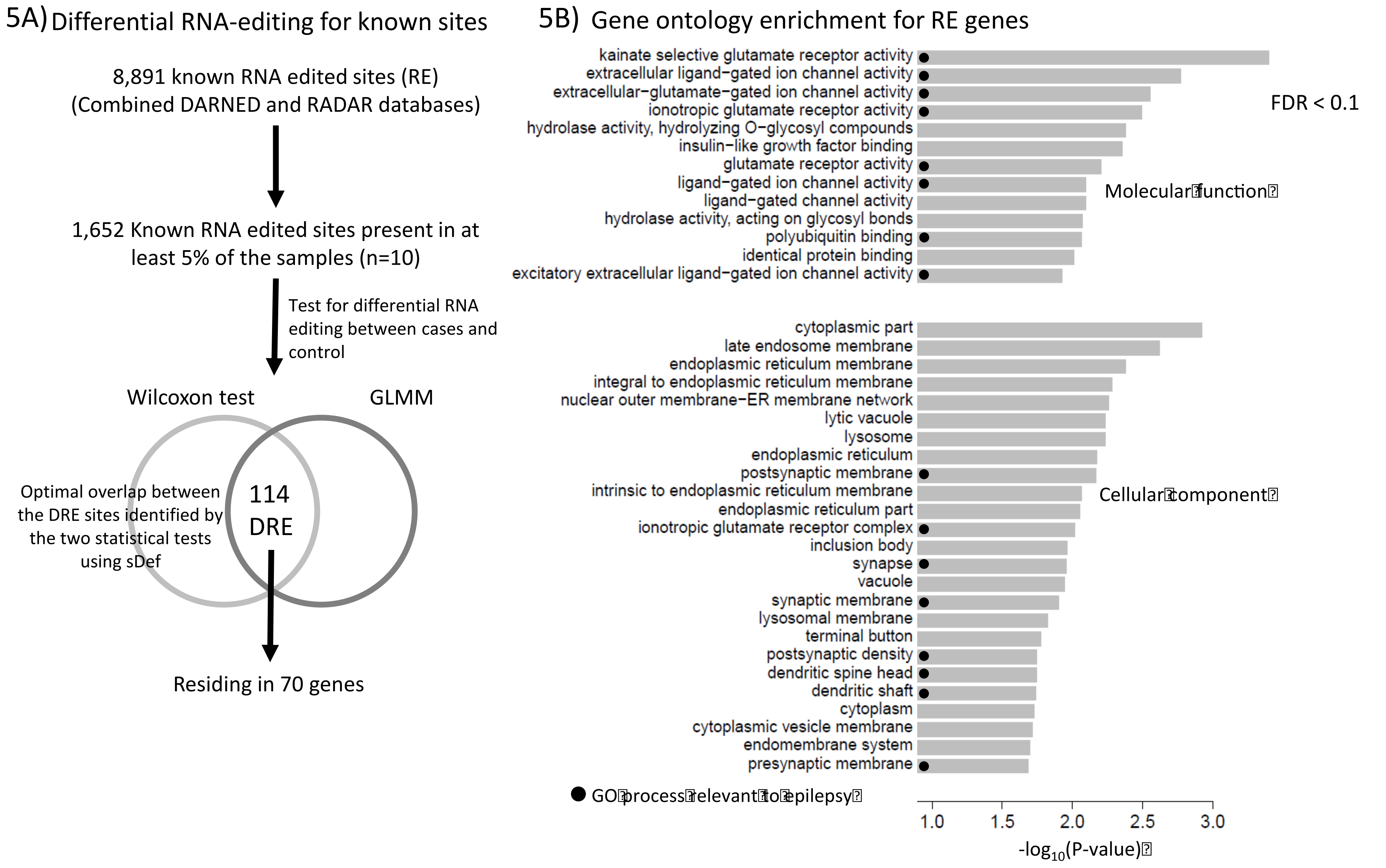

Supplementary Figure 5: Characterization of previously reported RNA-edited sites A**)** Differential RNA edited sites estimated from the two methods, **B**) Functional enrichment of the genes with differential RNA editing events by considering expressed genes as background

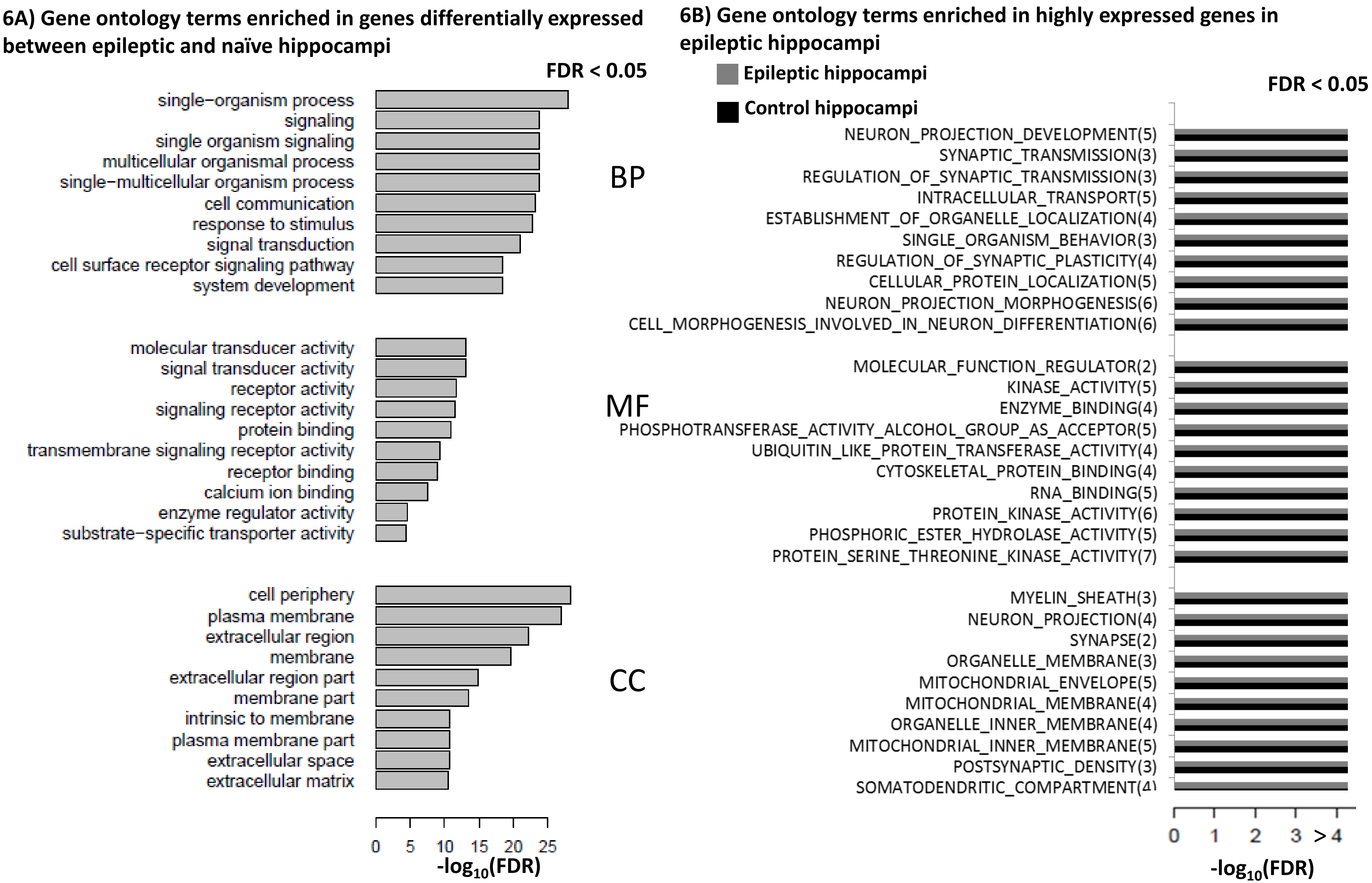

Supplementary Figure 6:A) Top 10 functional enrichment of the differentially expressed genes by considering expressed genes as background. **B)** Top 10 functional enrichment of the genes that were highly expressed in epileptic (grey bar) and control mice (black bar) . The GO enrichment analysis was performed used GSEA approach with 10,000 permutation so the P-value we cannot be less than 10^{-5} Therefore on X-axis maximum value for –log10(P-value) is represented as >5. The results are summarized for following gene ontology categories Biological processes (BP), Molecular function (MF) and cellular components (CC).

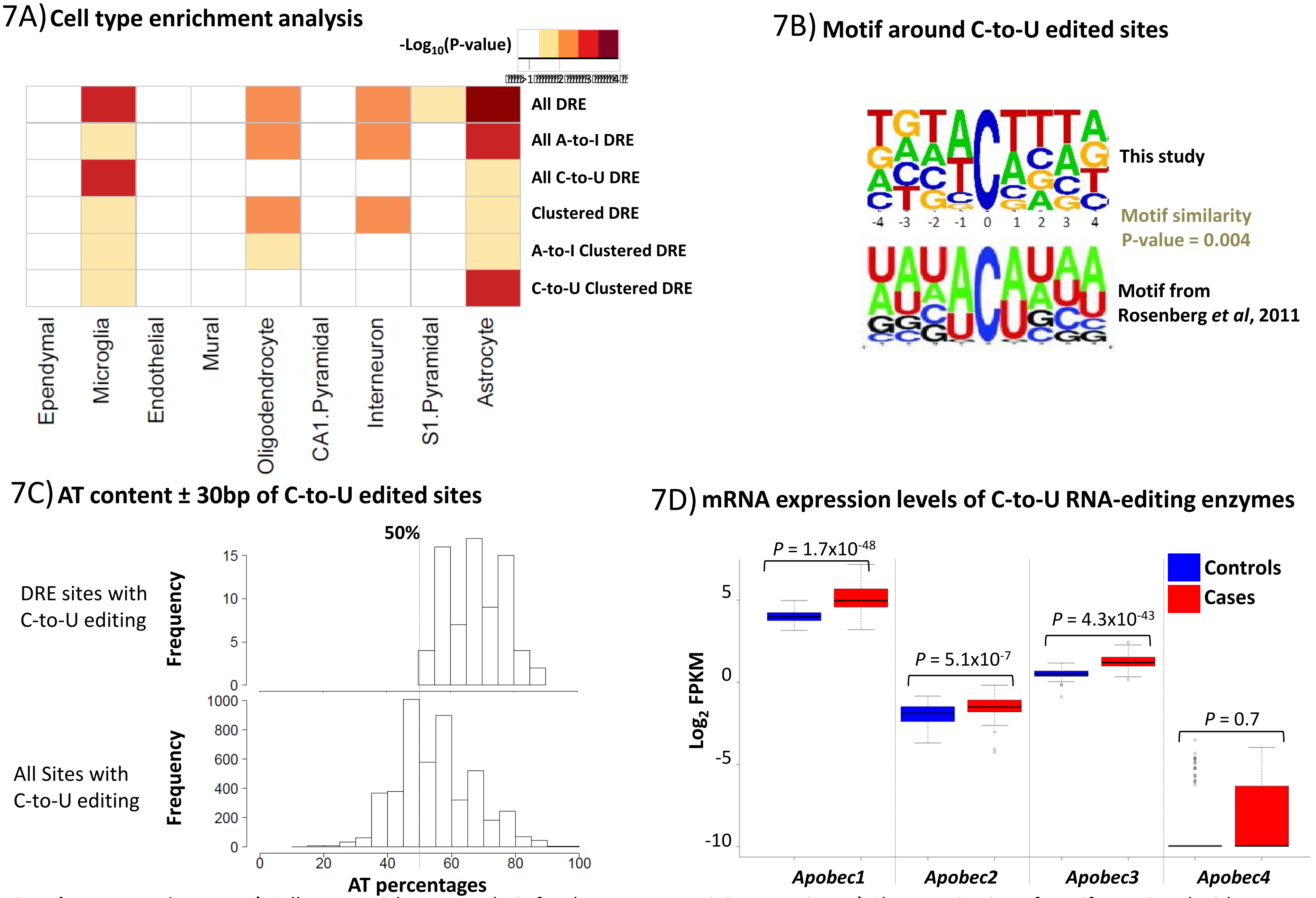

Supplementary Figure 7: **A)** Cell type enrichment analysis for the genes containing DRE sites **B)** Characterization of motif associated with non-clustered differential RNA edited sites, Top shows AT content around C-to-T edited site, **C)** Characterization of AT content associated with C-to-T RNA edited Top shows AT content around differential C-to-T edited site Bottom shows AT content around all predicted C-to-T edited sites. **D)** Gene expression differences for the key gene coding C-to-T RNA editing enzyme i.e. Apobec family

Supplementary Figure 8:

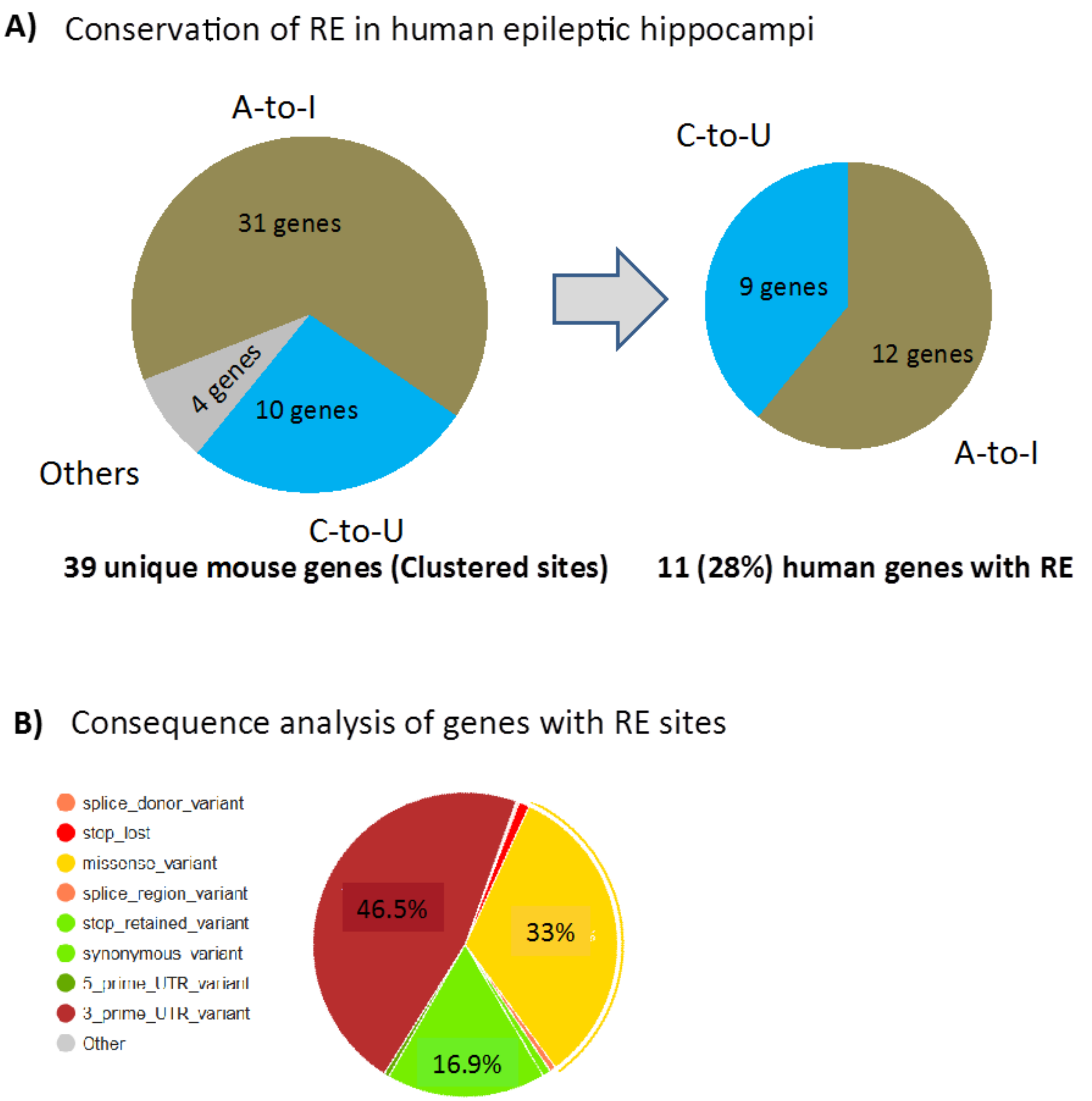

Supplementary Figure 8: **Conservation of RE in genes with differential RNA editing events in human epileptic hippocampi. A)** A figure showing degree of conservation between differential RNA-edited sites in human epileptic hippocampi. **B)** Consequence analysis of conserved RNA-edited sites in human epileptic hippocampi.
