# Supplementary material for: Genome-wide analysis of differential RNA editing in epilepsy
Source: Genome Res. 2017 Mar;27(3):440–50. doi: 10.1101/gr.210740.116 (PMC5340971; doi:10.1101/gr.210740.116)
Supplement: Supplemental Material [file supp_gr.210740.116_Supplementary_Table_S7.docx]

**Supplementary** **Table 8.** Genes with DRE in mouse hippocampus that are conserved in human epileptic hippocampus.
