# Supplementary material for: Genome-wide analysis of differential RNA editing in epilepsy
Source: Genome Res. 2017 Mar;27(3):440–50. doi: 10.1101/gr.210740.116 (PMC5340971; doi:10.1101/gr.210740.116)
Supplement: Supplemental Material [file supp_gr.210740.116_Supplementary_Text.docx]

**Supplementary Text 1**

**Simulating RNA-editing percentages**

To estimate the effect of ‘noise’, which might be due to a high proportion of non-canonical RE sites in the RE screen, we first simulated RE percentages for 50 cases and controls based on data-based non-parametric method (Benidt and Nettleton 2015). This data-based simulation involved subsampling from a large population (case/control) in such a way that the RE distribution and the null hypothesis of no difference in mean RNA-editing percentage between the two groups is satisfied, except for a set of differential RE sites. The subsampling procedure in 100 cases and 100 controls was performed using an R package SimSeq (Benidt and Nettleton 2015), and generated 10 bootstrap samples. Next, we added different noise levels to each of the simulated bootstrap samples, using two models, as follows:

*Model-1*: For each edited site, the simulated RE percentages (X) were obtained by adding or subtracting at random a fixed percentage (Y) of initial RE level:

X=RE level ± Y (1)

Y is the percentage of RE level ∈ {50%, 75%, 100%, 200% and 300%} and X is the simulated RE percentage with noise.

*Model-2*: For each edited site, the simulated RE percentages (X’) were obtained by adding or subtracting at random a percentage (Y’) of initial RE level, where Y’ is drawn from a normal distribution with mean X (as in (1)) and standard deviation (SD) as a function of mean, as described below

X’=RE level ± Y’

Y’ is percentage of RE level ~ N (mean=X & SD ∈ {0.5X, 0.75X, X, 2X, 3X, 4X, 5X and 10X}) and X=RE level ± Y from (1).

This generates a total of (5 x 8 noise levels) x (10 bootstrap samples) x ((50 cases) + (50 controls)) = 400,000 data points that constitute the datasets of RE levels (with varying noise) simulated in 50 cases and 50 controls.

**Estimating true/false positive rate in the DRE screen**

To estimate true/false positive rate in the differential RNA editing analysis, fifty cases and fifty controls were simulated using data-based non-parametric (see above for detail). These simulated datasets retain key features of the real data that were used in our study, and the mean and variance for respective RE sites were comparable to the original non-simulated cases and controls datasets (Suppl. Fig. I).

Within each simulated dataset (cases or controls), varying noise levels were added to a random proportion of RE sites: 10, 15, 20, 25, 30, 35, 40 and 45% all RNA sites. This yields several datasets for which the noise affects different fractions of the data, ranging from a small proportion of RE sites (10%) to almost half of the RE sites (45%). We then carried out the differential RE analysis (using Wilcox rank test, in keeping with the real data analysis presented in the manuscript) and calculated the number of correctly recalled DE sites (true positive, TP). As expected, for all the simulated scenarios the TP rate decreases linearly as a function of the percentage of noise added and the proportion of sites to which noise is added (see Supplemental Fig. II for representative examples). We then analysed all results and extrapolated the expected proportion of false positives (FP) in the DRE screen for the worst-case simulated scenario, showing that maximum FP rate is around 10% (depending on the specific choice of noise model).

Since the percentage of non-canonical sites in our study for ‘All’ edited sites (9,277) was 77.9% (A/G + C/T site fractions), the non-canonical sites observed are ‘noise’ then the estimated true positive rate according to non-canonical percentages in DRE would be ~90% for ‘All’ datasets respectively. This is very close to the percentages observed in ‘All’ DRE sets, which is 92.8 %. Thus suggesting that potential false positive rate would be around 10% depending on the respective choice of noise model and percentages of sites with noise (Supplemental Fig. II).

| 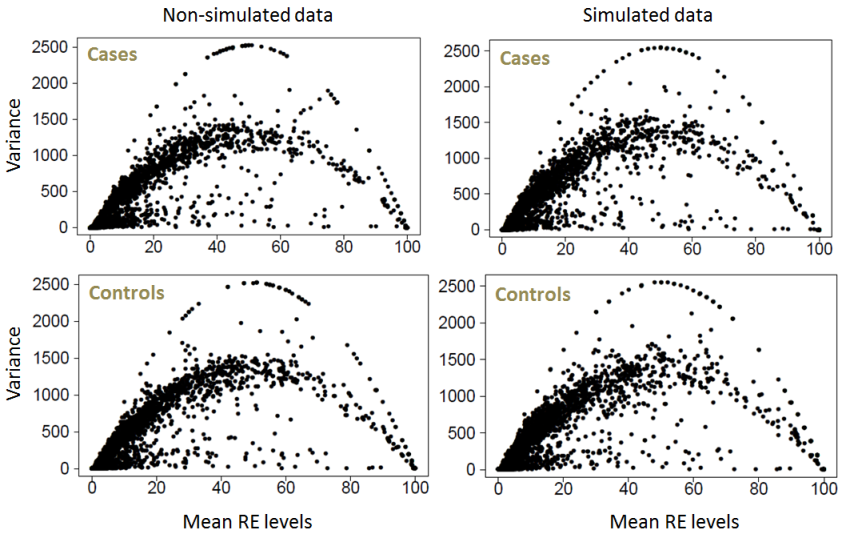 | 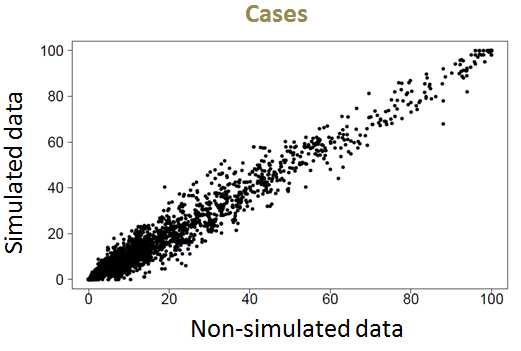  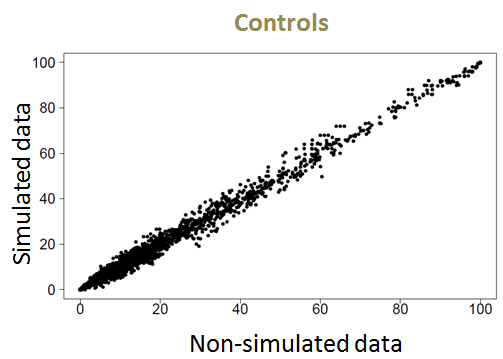 |
| --- | --- |
| **Suppl. Fig. I:** Mean and variance are plotted against each other to compare simulated dataset with real dataset in epileptic and control hippocampus (left panel). We also compared mean editing levels for simulated data with real dataset for both epileptic and control hippocampus (right panel). These plots highlight that the simulated and real datasets are consistent in their mean RE levels and their variances. | |

|  |
| --- |
| **Suppl. Fig. II:** The figure summarises the results obtained after simulating RE percentages, the noise was added to simulated datasets systematically by considering different models of noise. In the panels, above results from different noise models are presented: Panel (A) summarizes results based on Model-1 noise model. Panels (B)-(I): summarizes results based on Model-2 where SD was = 0.5 times mean (B), = 0.75 times mean (C), = mean (D), = twice value of mean (E), = three times value of mean (F), = four times value of mean (G), = five times value of mean (H) or = Ten times the value of mean (I). The percentages of non-canonical sites before performing differential RE analysis was 22% and 15% for the list of ‘All’ and ‘clustered’ sites respectively. To assess the potential confounding effect of non-canonical RE sites on the differential analysis, we carried out a simulation study where the noise is affecting ~20-25% (highlighted in grey in the plots above) of the sites. We estimated that the false positive rate associated with the ‘differential’ RNA-edited sites is ~10% for unclustered ‘All’ RE sites and ~5% for ‘clustered’ RE sites, respectively. |

**References:**

Benidt S, Nettleton D. 2015. SimSeq: a nonparametric approach to simulation of RNA-sequence datasets. *Bioinformatics* **31**: 2131-2140.
